# The functional impact of home-based self-rehabilitation following arthroscopic meniscus root repair

**DOI:** 10.1186/s12891-022-05662-6

**Published:** 2022-08-05

**Authors:** Mohammad Tahami, Arash Sharafat Vaziri, Mohammad Naghi Tahmasebi, Mohammad Amin Ahmadi, Armin Akbarzadeh, Fardis Vosoughi

**Affiliations:** 1 Bone and Joint Diseases Research Center, Department of Orthopedic Surgery, Chamran Hospital, Shiraz University of Medical Sciences, Shiraz, Iran; 2 Department of Orthopedic and Trauma Surgery, Shariati hospital, Tehran University of Medical Sciences, Tehran, Iran; 3 Center of Orthopedic Trans-Disciplinary Applied Research (COTAR), Shariati Hospital, Tehran University of Medical Sciences, Tehran, Iran

**Keywords:** Arthroscopy, Rehabilitation, Meniscus, Lysholm, Postoperative, Function, COVID, Corona virus

## Abstract

**Background:**

Corona virus infectious pandemic makes outdoors rehabilitation a potential hazard. Patient education to perform simple home-based exercises seems to be an interesting and sometimes a mandatory option. This study provides a comparison between the conventional and home-based virtual rehabilitation after surgical repair of medial meniscus root tears.

**Methods:**

In this prospective study, all patients who underwent medial meniscus posterior root repair with a modified trans-tibial pull-out technique from March 2019 to March 2021 were evaluated. Those who underwent surgery after December 2019 were trained to perform self-rehabilitation. The rest had undergone outdoors specialized rehabilitation according to a unified protocol and these were used as a historical control group. All patients were followed up for a minimum of 2 year after surgery. Final Lysholm scores were utilized to compare functional outcomes after considering the effect of age, body mass index and time from surgery by multivariate linear regression analysis.

**Results:**

Forty-three consecutive patients with medial meniscal root tears were studied. Thirty-nine (90.7%) were women and 4 (9.3%) were men. The mean age of participants was 53.2 ± 8.1 years. The total Lysholm knee score, and all its items were significantly improved in both groups at a two-year follow-up (*p* < 0.05), except the “Using cane or crutches” item (*p* = 0.065). Nevertheless, the final Lysholm knee score improvement was higher in patients who performed outdoors specialized rehabilitation and in patients with shorter time-to-surgery.

**Conclusion:**

Regardless of age and gender, home-based rehabilitation after meniscal root repair with the modified trans-tibial pull-out technique improved the patients’ function at a two-year follow-up. Nonetheless, this effect was still significantly lower than that of the outdoors specialized rehabilitation. Future work is required to clarify basic protocols for home-based tele-rehabilitation programs and determine clinical, radiological and functional results.

**Level of evidence:**

Level IV, therapeutic, historically controlled study.

## Introduction

Meniscal roots convert the axial load into hoop stress and distribute the pressure symmetrically in the articular surface [1]. Root injuries are defined as either avulsion of posterior tibial attachment or radial tear of the posterior horn within 1 cm of its attachment [2, 3]. The meniscal root injury results in meniscal extrusion and if left untreated, root injury can lead to early osteoarthritis [4–8]. Several methods have been introduced for diagnosis and repair of a medial meniscal posterior root tear [9–11]. The trans-tibial pull-out repair technique involves passing a suture through the meniscal root and retrieving it through a tibial tunnel. Screw or button fixation can then be used. Biomechanical and clinical outcomes of different suturing techniques have been previously scrutinized [12–14]. Despite recent advances in meniscal root repair, this remains a challenging procedure with several potential complications including loosening and re-tear [15, 16].

The effects of corona virus infectious disease (COVID-19) pandemic on orthopedics cannot be overlooked [17–19]. It has profoundly affected postoperative rehabilitation. COVID-19 pandemic has created further obstacles on the way of achieving the best possible functional outcomes. Most patients are afraid to participate in outdoors rehabilitation and cannot afford home-based private physical therapy.

In the current study, patients with medial meniscal posterior root tear (MPRT) underwent surgical repair of the tear with a modified trans-tibial pull-out technique. We sought out to determine (1) if these patients can experience significant improvement in function with home-based self-rehabilitation, and (2) if there is a significant difference in functional outcomes between the patients who are forced to perform home-based self-rehabilitation and those who have access to specialized physical therapy.

## Patients and methods

This retrospective cohort studies all patients who underwent surgical repair of the MPRT from March 2019 till March 2020 in a tertiary knee center. The study protocol was reviewed and approved by the local ethics committee. The procedure was described for all patients and informed written consents were obtained. Two separate fellowship trained knee surgeons were involved who used the same surgical technique for root repair. Baseline patient characteristics and the time interval from the acute onset/exacerbation of knee pain to the surgery (time-to-surgery intervals) were recorded. The severity of knee osteoarthritis prior to and after surgery was assessed based on Kellgren- Lawrence (K-L) classification [20]. Surgical repair was considered for patients with symptomatic MPRT with a stable knee joint and no major malalignment or severe osteoarthritis (KL II or less). Diagnosis of an MPRT was confirmed with magnetic resonance (MR) imaging, after identifying relevant clinical findings [2]. Those patients younger than 18 years of age, those with less than two-year follow-up or with concomitant anterior cruciate ligament (ACL) injury were excluded from the study. Those patients who underwent surgery after December 2019 (COVID-19 era patients) were trained to perform self-rehabilitation. Those who underwent surgery and completed their rehabilitation before December 2019 (non-COVID era patients) had undergone outdoors specialized rehabilitation according to a unified protocol and these were used as a historical control group. This methodology is sound and has been used before [21]. Patients were examined for a follow-up period of at least two years after surgery by their surgeon, and Lysholm knee score was recorded [22].

### Surgical technique

**Fig. 1:**
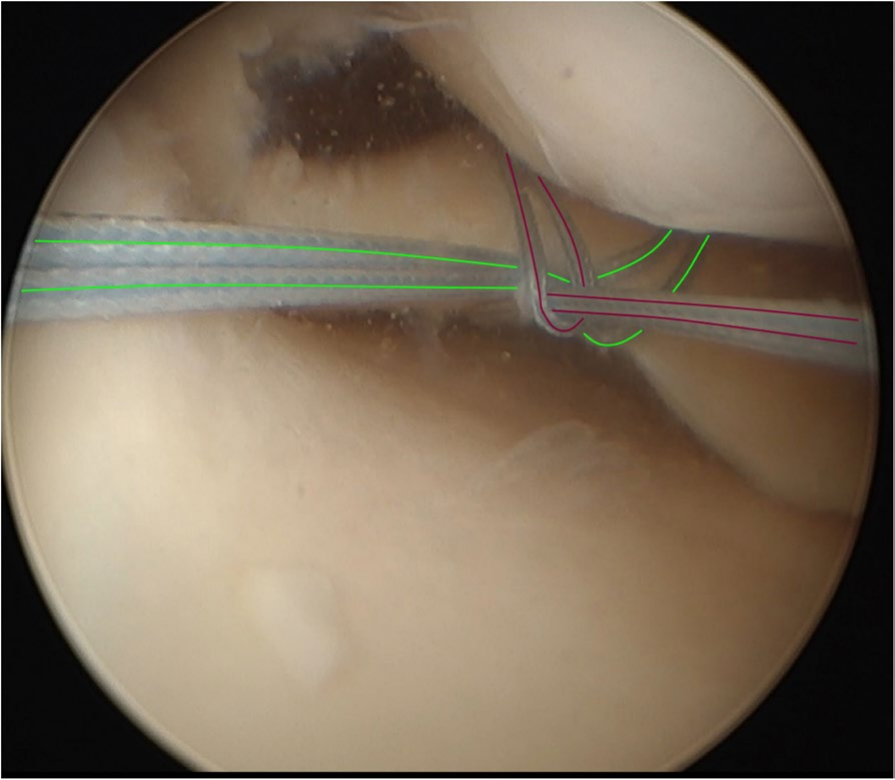
The arthroscopic view of the Loop-Post Construct technique. The first and second loop constructs are identified by green and red lines, respectively [23]. (Reproduced with permission from: Tahami M, Vaziri AS, Tahmasebi MN. Loop-Post Construct, A Novel Technique for Medial Meniscal Root Repair. Archives of Bone and Joint Surgery. 2020;8(4):545.)

The loop-post construct technique, which was introduced in 2020, as a modification of the standard trans-tibial pull-out method of repairing meniscal root tears was used [23]. After performing a diagnostic arthroscopy via the anterolateral (AL) portal, the near anteromedial (AM) portal was created by a vertical incision just adjacent to the medial border of the patellar tendon. Notchplasty of the medial wall helped to provide better access to the MPRT in cases of a narrow notch. Percutaneous release of the superficial medial collateral ligament was performed in all cases to increase the working space. A far AM portal was then created by a horizontal incision after identifying the appropriate location using a spinal needle. The MPRT footprint was identified and freshened using a curette. The meniscal root was reduced by an arthroscopic grasper. If scar tissue or fibrosis was limiting the mobility of the meniscus, it was debrided to release the meniscal root and help its reduction into the footprint. The fibrotic end of the torn or avulsed meniscal root was freshened with a shaver. Through the far AM portal, the EZPass™ 70˚ Suture Passer (Zimmer-Biomet) was introduced. A nylon 1/0 thread was passed from the superior to the inferior surface of the meniscus one centimeter from the torn end as a shuttle, to help passing the Fiber Wire 2–0 suture (Arthrex, Naples, FL) or Express-Braid™ no.2 suture (Zimmer Biomet, Warsaw, IN) as the first loop. The second loop was created in a similar manner at 5 mm from the torn end of the meniscal root (in the traditional trans-tibial pull-out technique, both sutures were passed 5 mm from the edge). Before tightening the second loop, both free ends of the first loop were passed and locked under the second loop and then they were retrieved through the portal (Fig. 1). In order to create the tibial tunnel, a tibial target guide for ACL (Karl Storz, Tuttlingen, Germany or Conmed Linvatec, USA) was used. The guide was inserted through the near AM portal, and its tip was placed at the footprint. Reaming was performed with a 4.3 mm ACL reamer. A Flip Cutter® II, 8^ mm^ Drill (Arthrex) was inserted through the reamed canal, to (1) confirm the tunnel’s position in the anatomical footprint with arthroscopy and (2) perform minimally invasive inside-out reaming of the tibial tunnel. Both ends of the loop and post constructs were retrieved through the tunnel. Tension was applied to the thread ends in 30˚ of knee flexion, and then they were fixed on the tibial cortex around a screw-washer construct. We tend to over-reduce the meniscus by tensioning the root to the point that at least 5 mm of the root enters the tunnel before final fixation.

### Postoperative rehabilitation

Rehabilitation protocols were adjusted from Mueller et al. meniscus root rehabilitation concepts [24]. The knee was immobilized and locked in extension for two weeks. Range of motion exercises started 2 weeks postop, with the goal of reaching 90 degrees of flexion by the end of the 6^th^ week. Passive range of motion exercises to reach 90 degrees of flexion during the first six weeks after the operation included supine wall slides and hanging the leg from the bed. Patellar mobilization exercises were started and performed by the therapist or the patient himself. Open chain quadriceps exercises were performed immediately after surgery during hospitalization period under supervision. Furthermore, partial weight-bearing exercises (i.e., toe touching using crutches) started during the first two weeks, with the brace locked in extension. Full weight-bearing was permitted after six weeks.

Postoperative rehabilitation was performed either as an outdoors specialized rehabilitation or by the patients themselves as a home-based self-rehabilitation due to the force of isolation after the COVID-19 outbreak. Self- rehabilitation at home included training of the patients to perform straight leg raising, range of motion and patellar mobilization exercises. Patient education was performed by the surgeon and the physical therapist before discharge from the hospital. After being discharged, virtual education and virtual follow-up with the physical therapist using social media, mostly Whatsapp (Facebook, Inc) helped to ensure proper adherence to the instructions and patient progressions during this process were supervised by both the surgeon and the physical therapist. The virtual postoperative follow ups were scheduled weekly until 6 weeks or until achievement of 90’ flexion and full weight bearing; whichever happened sooner. After which the patient was followed virtually at 3 months postoperative.

Meanwhile, outdoors specialized rehabilitation was performed by a trained physical therapist with a uniform protocol including range of motion exercises, vastus medialis strengthening, patellar mobilization, open-chain quadriceps isometric exercises, hamstring stretching and pain reduction modalities.

### Statistical analysis

Statistical analysis was applied by R programming language (version 3.3.1 for Mac OS) with deducer graphical user interface (GUI) package, and the results were visualized by GraphPad Prism (version 8.2.1 for Mac OS). Quantitative and qualitative variables were described using mean ± standard deviation (SD) or median and frequency (percentage), respectively. The primary objective was to compare the baseline and the two-year post-surgical total Lysholm knee score and its domains. The effect of demographic, clinical variables and type of rehabilitation on Lysholm Knee Score change (Δ LKS) was analyzed using the Wilcoxon rank-sum test. Comparing means for normally distributed variables was performed by paired t test. Correlations were test by the Mann–Whitney test. A p-value < 0.05 was considered as statistically significant. Multivariate linear regression analysis was performed to detect the effect of age, BMI, time from surgery and type of rehabilitation on final LKS.

## Results

**Fig. 2:**
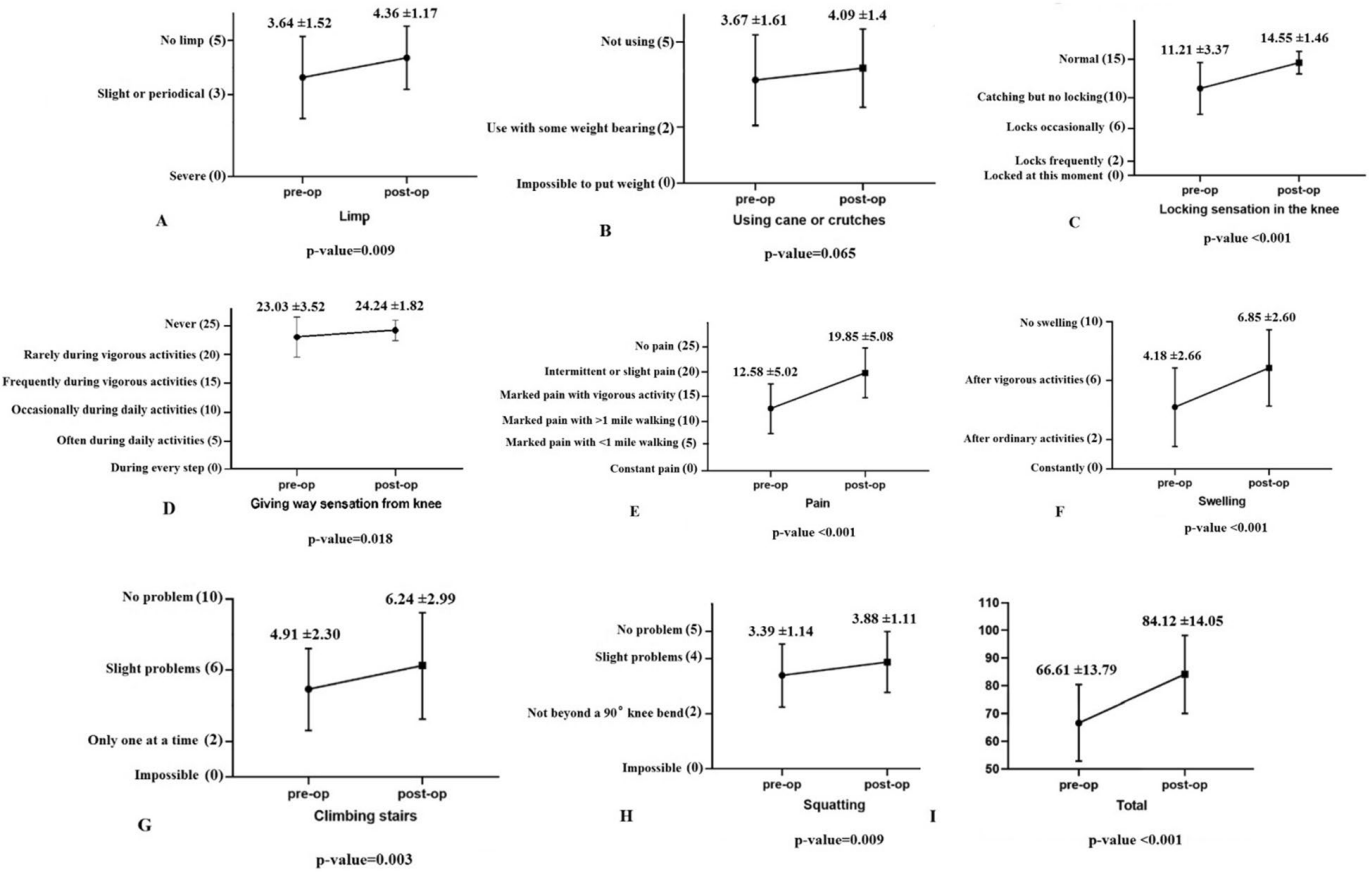
The Lysholm knee score of Pre-operative and two-year post-operative follow-up of the patients who treated with the Loop-post Construct technique for medial meniscal root repair

Forty-nine consecutive patients who underwent root repair with the modified trans-tibial pull-out technique during the specified time period were eligible for inclusion in the study. Six patients were excluded (lost to follow-up), leaving 43 patients who participated in this study. Patient characteristics and clinical outcomes are provided separately (Tables 1 and 2). Approximately, two-thirds of the patients completed outdoors specialized rehabilitation before the COVID-19 outbreak (29 [67.4%]). The remaining 14 patients [32.5%] were trained to perform home-based self-rehabilitation. The median Kellgren- Lawrence grades of knee osteoarthritis were 1 both prior to and two years after surgery. No patient experienced a change in the grade of osteoarthritis during the study time frame. Furthermore, according to the total Lysholm knee scores, two-year functional outcome was excellent in 16 (37.2%), good in 18 (41.8%), fair in 7 (16.2%) and poor in 2 (4.6%) patients. Figure 2 illustrates pre-operative and two-year post-operative functional scores of patients using the Lysholm knee score (the entire cohort). The total final Lysholm knee score (LKS), along with all its subscales showed significant improvement in both groups, except the “Using cane or crutches” subscale which showed no significant difference (*p* = 0.065) (Fig. 2).

**Table 1:** Baseline characteristics of the patients who underwent medial meniscal root repair with Loop-Post construct technique

Variable, *N* = 43	Value
Age, mean (SD)	53.2 (8.1)
Sex, N (%)	
Female	39 (90.7)
Male	4 (9.3)
Time to surgery, months	5.24 (2.89)
BMI, mean (SD)	28.14 (2.02)
Specialized rehabilitation (non-COVID era), N (%)	29 (67.4%)
Kellgren- Lawrence grade prior to surgery, median	1 (0–2)
Kellgren- Lawrence grade after surgery, median	1 (0–2)

*N*: number, *SD* Standard Deviation, *BMI* Body mass index (Kg/m^2^), *CI* Confidence interval

**Table 2:** Clinical outcomes of the patients who underwent medial meniscal root repair with Loop-Post construct technique

Variable	Value	p-value
Lysholm knee total score, N (%)		
Excellent	16 (37.2)	
Good	18 (41.8)	
Fair	7 (16.2)	
Poor	2 (4.6%)	
Lysholm knee score reduction (improvement)		
Rehabilitation		
Specialized rehabilitation (non-COVID era)	20 ± 9.13	0.012
Home-based self-rehabilitation (COVID era)	12.55 ± 5.01	
time to surgery, *r* coefficient (95%CI)	-0.51 (-0.73, -0.20)	0.002
Sex		
male	15.51 ± 4.34	0.802
female	18.16 ± 9.65	
Age, *r* coefficient (95%CI)	0.045 (0.30,0.38)	0.849

**Fig. 3:**
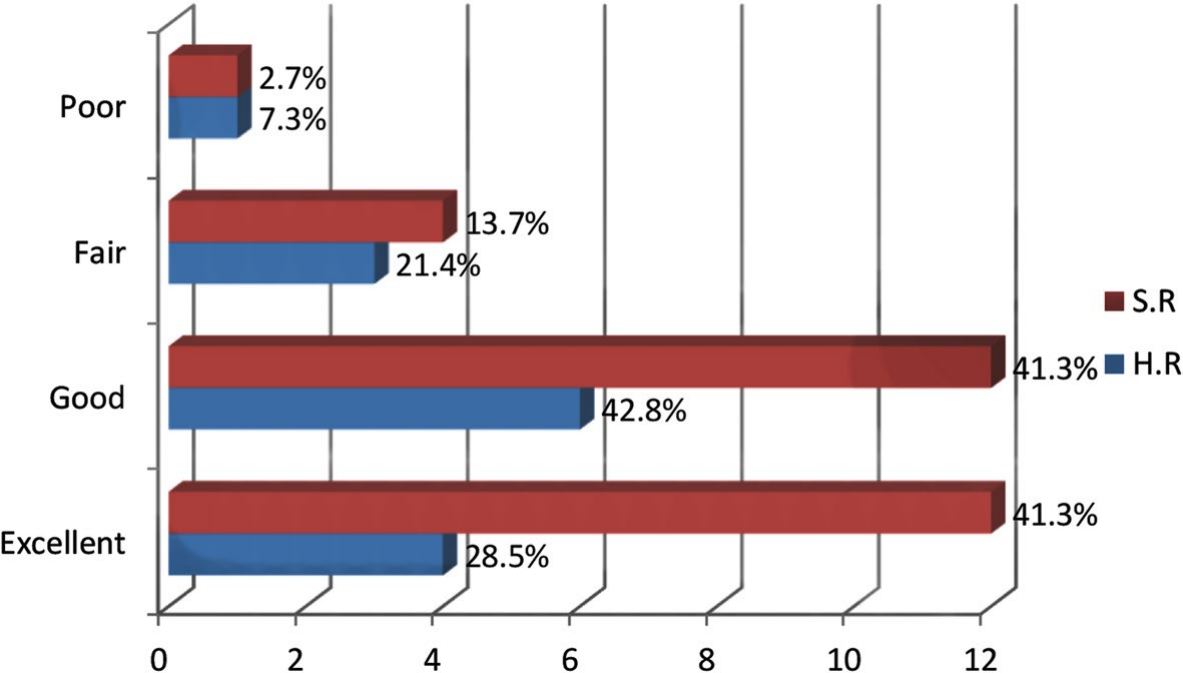
Comparison between the patients’ final self-reported functional status in the specialized rehabilitation (SR) and home-based rehabilitation (HR) groups

The increase in the final Lysholm knee score (which means functional improvement) was higher in patients in the non-COVID era (Δ LKS _+rehabilitation_ = 20 ± 9.13 vs, Δ LKS _-rehabilitation_ = 12.55 ± 5.01; *p* = 0.012) (Fig. 3). Furthermore, in both groups, improvement was significantly higher among the patients with shorter time-to-surgery interval (*r* coefficient = -0.51, 95%, confidence interval (CI) = -0.7264, -0.2026; *p* = 0.002). Patient’s age (*r* coefficient = 0.045, 95% CI = -0.3027, 0.3827; *P* = 0.849) and sex (Δ LKS _female_ = 18.16 ± 9.65 vs. Δ LKS _male_ = 15.5 ± 4.34; *P* = 0.802), on the other hand, did not show any significant correlation with final Lysholm knee scores.

Multivariate analysis (Table 3) showed that, after considering the confounding effect of age, BMI and time from surgery, still the type of rehabilitation was an independent factor associated with higher final Lysholm scores (β = 7.6, *P* = 0.008).

**Table 3:** Multivariate linear regression analysis to determine the effect of age, body mass index (BMI), time (from surgery) and type of rehabilitation on final Lysholm knee scores (LKS)

	Beta	*P* value
Constant	28.890	
BMI	-0.332	0.183
Rehabilitation	-7.631	0.614
age	0.179	0.008
time	-0.270	0.541

## Discussion

During the last 15 years, techniques for surgical repair of MPRT have been developed to restore joint biomechanics and joint contact pressures, to prevent the joint from early osteoarthritis. Trans-tibial pull-out repair is one of the most common procedures for meniscal root repair [25]. The transtibial tunnel drilling releases growth factors and progenitor cells from bone marrow and may improve the healing process [26]. Moreover, previous studies [15, 27] reported significant improvement in clinical and radiographic outcomes. A slight modification of the trans-tibial pull-out technique has recently been introduced, called the loop-post construct technique, for meniscal root repair [23]. In the present study, the two-year functional outcomes of this technique showed significant improvement in Lysholm knee scoring items. Admittedly, without a control group utilizing a standard trans-tibial pull-out technique, it seems inappropriate to conclude anything on the clinical advantage of this modified technique.

Included patients were mostly females at their mid-fifties. Patients with a stable knee and no sign of severe osteoarthritis or major malalignment were scheduled for surgical repair of the MPRT. This demographic pattern and surgical indications are in consistency with previous reports [28, 29]. Conversion to total knee arthroplasty and the progression of Kellgren-Lawrence grade are two noticeable concerns in choosing the treatment modality for meniscal root tear. Hence, root repair is a wise choice for active patients with acute root tears or those with root tears who have minimal or absent osteoarthritis [14, 26, 30, 31]. Noticeably, our results show no progression of osteoarthritis during the study time frame; nevertheless, long-term follow up periods might yield different results.

No correlation was seen between demographic characteristics such as age or gender and functional outcomes after root repair. This finding is interesting since we expected to see variable results of self-rehabilitation between different age/sex groups, due to different levels of compliance. Frankly this study involved a limited age range, which might have masked this effect. Besides, previous studies such as Laprade et al. [32] reported no significant differences in clinical and radiological changes between patients older than 50 and younger than 50 years of age. The effects of age and gender on the functional outcomes of home-based rehabilitation have yet to be proven.

Time interval from the onset/ exacerbation of knee pain to surgery was significantly correlated with clinical outcomes. As a result, assigning meniscal root repair methods as soon as possible amongst eligible patients might improve the outcomes of the surgery, a notion which has been stated in the previous reports [2].

All patient undergoing root repair in our centers were started on a specialized rehabilitation program with a unified protocol in the non-COVID era. Outbreak of the SARS-COV-2 infection in December 2019 and isolation protocols prevented the patients from participating in such programs. Most patients were afraid to perform outdoors rehabilitation and were unable to afford home-based private physical therapy either. Therefore, we were forced to train the patients to perform simple rehabilitation tasks at home.

Our results show significant improvement in patient reported outcomes in both non-COVID and COVID era patients. Fortunately, no case of limited knee range of motion was encountered in neither group. Still and all, those who had access to specialized physical therapy (the non-COVID era group) experienced significantly better outcomes. While no study was found in the literature after the COVID-19 outbreak to take this matter into consideration, some previous reports have considered a comparison between restricted and accelerated rehabilitation [33–35]. VanderHave et al. [33], for instance, found a comparable successful clinical outcome regarding restricted and accelerated rehabilitation (70–94 vs. 64–96%). On the other hand, Vascellari et al. [34] did not report a difference in repair failure (10% vs. 13%). Noticeably, significant heterogeneity existed among previous reports.

Before jumping into any conclusions, one must consider some serious limitations of this study. We did not use visual analogue scale for pain, however we reported Lysholm scores which show the level of pain patients encounter during everyday activities. Lack of MRI evaluation and a follow-up of two years are two important limitations of this study. The COVID-19 outbreak has only begun since two to three years ago; therefore, follow-up period could not be any longer and our sample size is relatively small. Even so, we felt compelled to share our concerns and results. Indeed, this seems to be a global on-going problem which might deeply affect not only our routine clinical practice, but also our rehabilitation protocols and postoperative care. Due to a lack of control subjects, we compared the results with historical controls who had completed their postoperative rehabilitation before the start of the pandemic. Another limitation, is the modification of the standard trans-tibial pull-out technique that we used to repair root tears [23]. No biomechanical testing has been performed for this technique, still, its basics have been proven both biomechanically and clinically in the literature [13, 14, 32].

## Conclusion

In summary, the results reveal that regardless of age and gender, patients can reach significant functional improvements even with home-based simple rehabilitation tasks after arthroscopic repair of MPRT. Nonetheless, better outcomes were associated with postoperative specialized rehabilitation programs and earlier surgery. Future work is required to clarify basic protocols for home-based tele-rehabilitation programs and determine clinical, radiological and functional results.

## Data Availability

All data generated or analyzed during this study are included in this published article.
